# Correction: Seroprevalence and longevity of SARS-CoV-2 nucleocapsid antigen-IgG among health care workers in a large COVID-19 public hospital in Saudi Arabia: A prospective cohort study

**DOI:** 10.1371/journal.pone.0305375

**Published:** 2024-06-06

**Authors:** The PLOS ONE Staff

There are errors in the author affiliations. The correct affiliations are as follows:

Faisal Alasmari^1,2^, Mahmoud Mukahal^1^, Alaa Ashraf Alqurashi^3^, Molla Huq^4^, Fatima Alabdrabalnabi^1^, Abdullah AlJurayyan^5^, Shymaa Moshobab Alkahtani^6^, Fatimah Salem Assari^6^, Rahaf Bashaweeh^6^, Rana Salam^7^, Solaf Aldera^1^, Ohud Mohammed Alkinani^8^, Talal Almutairi^9^, Kholoud AlEnizi^10^, Imad Tleyjeh^2^

**1** Infection Control and Environmental Health Administration, King Fahad Medical City, Riyadh, Saudi Arabia, **2** Infectious Diseases Section, King Fahad Medical City, Riyadh, Saudi Arabia; College of Medicine, Al Faisal University, Riyadh, Saudi Arabia, **3** Research Center, King Fahad Medical City, Riyadh, Saudi Arabia, **4** Department of Epidemiology and Preventive Medicine, Monash University, Melbourne, Australia, **5** Immunology and Serology Laboratory, King Fahad Medical City, Riyadh, Saudi Arabia, **6** College of Medicine, Al Faisal University, Riyadh, Saudi Arabia, **7** Public Health College, Saudi Electronic University, Riyadh, Saudi Arabia, **8** Infectious Diseases Section, King Fahad Medical City, Riyadh, Saudi Arabia, **9** Pathology and Clinical Laboratory Administration, King Fahad Medical City, Riyadh, Saudi Arabia, **10** Radiology Service Administration, King Fahad Medical City, Riyadh, Saudi Arabia

The publisher apologizes for the error.
