# Supplementary material for: Seroprevalence and longevity of SARS-CoV-2 nucleocapsid antigen-IgG among health care workers in a large COVID-19 public hospital in Saudi Arabia: A prospective cohort study
Source: PLoS One. 2022 Aug 12;17(8):e0272818. doi: 10.1371/journal.pone.0272818 (PMC9374211; doi:10.1371/journal.pone.0272818)
Supplement: Attachment — Submitted filename: Review Manuscript PLOS USP (2).docx [file pone.0272818.s001.docx]

My appreciation goes for the article. Here are some points to note:

1. For writing techniques, please refer to the publisher formats and articles that have been published. For example: the abstract should also contain the methods, the essential results, and conclusions in one complete paragraph; the second paragraph in line 93-95 one paragraph consists only 1 sentence.
2. **In the abstract:**
(i) Important results from statistical surveys, and results from multivariable logistic regression should be added.
(ii) The main results of the demographic statistics (Table 1) that specifically consider three factors: IgG seropositive status, clinical, and epidemiological respectively, should also be included.
3. **In the methods:**
(i) HCW should be associated with the location where they work. It must be mapped what are four hospitals in one city look like, such as the capacity, etc. (related with line 117)
(ii) Statictics methods should be explain more clearly particularly in multivariate logistic regression. *Note:* Is the multivariable logistic regression same with the multivariate logistic regression?
(iii) Alpha used is too large. It should be 0.01.
4. **In the results:**
(i) Please show the multivariable (multiple) logistic regression model, follows by the estimate parameters, chi-square table, etc. The example of mathematical model is as follows:

(ii) The descriptive statistics is more attractive and interesting if it is represented by figure/graph, for example:

B: Proportion of SARAS-Cov-2 samples across cities and regencies in West Java, Indonesia.

C: Distribution of SARS-CoV-2 sequenced samples across different cities and regencies in West Java Indonesia.

D: Demography of samples.

Source: Azzania, et al. (2021). Analysis of SARS-CoV-2 Genomes from West Java, Indonesia. Viruses (13), 2097.

(iii) Comparisons should suffice in unrelenting sentences. (What is the comparison table for?)
(iv) Demographics should be linked to where they live.
(v) Add an analysis of the effects of gender on that demographic, i. e. the effect of these results (3 factors: IgG seropositive status, clinical, and epidemiological) on each sex.
