# Supplementary material for: Seroprevalence and longevity of SARS-CoV-2 nucleocapsid antigen-IgG among health care workers in a large COVID-19 public hospital in Saudi Arabia: A prospective cohort study
Source: PLoS One. 2022 Aug 12;17(8):e0272818. doi: 10.1371/journal.pone.0272818 (PMC9374211; doi:10.1371/journal.pone.0272818)
Supplement: Attachment — Submitted filename: Response to Reviewers.docx [file pone.0272818.s002.docx]

To: Asep K. Supriatna, Ph.D
Academic Editor
PLOS ONE

Re: Revision to PONE-D-21-40113

Title: Seroprevalence and longevity of SARS-CoV-2 nucleocapsid antigen-IgG among health care workers in a large COVID-19 public hospital in Saudi Arabia: A prospective cohort study

We would like to thank the Editorial Office and the reviewers for their time and valuable comments. We appreciate your efforts in critically reviewing our manuscript and we believe these suggestions would add more depth to our manuscript. We hope we have addressed your concerns appropriately. We look forward to seeing our manuscript published in PLOS ONE. Please find our response to your queries below:

| Academic editor | Response |
| --- | --- |
| 1. Please ensure that your manuscript meets PLOS ONE's style requirements, including those for file naming. | 1. DONE |
| In your Data Availability statement, you have not specified where the minimal data set underlying the results described in your manuscript can be found. PLOS defines a study's minimal data set as the underlying data used to reach the conclusions drawn in the manuscript and any additional data required to replicate the reported study findings in their entirety. All PLOS journals require that the minimal data set be made fully available. For more information about our data policy, please see <http://journals.plos.org/plosone/s/data-availability>.  Upon re-submitting your revised manuscript, please upload your study’s minimal underlying data set as either Supporting Information files or to a stable, public repository and include the relevant URLs, DOIs, or accession numbers within your revised cover letter. For a list of acceptable repositories, please see <http://journals.plos.org/plosone/s/data-availability#loc-recommended-repositories>. Any potentially identifying patient information must be fully anonymized.  Important: If there are ethical or legal restrictions to sharing your data publicly, please explain these restrictions in detail. Please see our guidelines for more information on what we consider unacceptable restrictions to publicly sharing data: <http://journals.plos.org/plosone/s/data-availability#loc-unacceptable-data-access-restrictions>. Note that it is not acceptable for the authors to be the sole named individuals responsible for ensuring data access.  We will update your Data Availability statement to reflect the information you provide in your cover letter. | The data underlying the results presented in the study are available from: irb1@kfmc.med.sa |
| We note that you have stated that you will provide repository information for your data at acceptance. Should your manuscript be accepted for publication, we will hold it until you provide the relevant accession numbers or DOIs necessary to access your data. If you wish to make changes to your Data Availability statement, please describe these changes in your cover letter and we will update your Data Availability statement to reflect the information you provide. |  |
| 4. PLOS requires an ORCID iD for the corresponding author in Editorial Manager on papers submitted after December 6th, 2016. Please ensure that you have an ORCID iD and that it is validated in Editorial Manager. To do this, go to ‘Update my Information’ (in the upper left-hand corner of the main menu), and click on the Fetch/Validate link next to the ORCID field. This will take you to the ORCID site and allow you to create a new iD or authenticate a pre-existing iD in Editorial Manager. Please see the following video for instructions on linking an ORCID iD to your Editorial Manager account: <https://www.youtube.com/watch?v=_xcclfuvtxQ> | 4. DONE |
| 5. Thank you for submitting the above manuscript to PLOS ONE. During our internal evaluation of the manuscript, we found significant text overlap between your submission and the following previously published works, some of which you are an author.  - https://www.medrxiv.org/content/10.1101/2020.07.09.20150136v1- <https://pubmed.ncbi.nlm.nih.gov/33341124/https://discovery.ucl.ac.uk/id/eprint/10117965/2/Walker_The%20duration%2C%20dynamics%20and%20determinants%20of%20SARS-CoV-2%20antibody%20responses%20in%20individual%20healthcare%20workers_AAM.pdf>  We would like to make you aware that copying extracts from previous publications, especially outside the methods section, word-for-word is unacceptable. In addition, the reproduction of text from published reports has implications for the copyright that may apply to the publications.  Please revise the manuscript to rephrase the duplicated text, cite your sources, and provide details as to how the current manuscript advances on previous work. Please note that further consideration is dependent on the submission of a manuscript that addresses these concerns about the overlap in text with published work.  We will carefully review your manuscript upon resubmission, so please ensure that your revision is thorough. | 5. DONE |
| Reviewer #1 |  |
| My appreciation goes for the article. Here are some points to note: 1.For writing techniques, please refer to the publisher formats and articles that have been published. For example: the abstract should also contain the methods, the essential results, and conclusions in one complete paragraph; the second paragraph in line 93-95 one paragraph consists only 1 sentence. | 1. DONE |
| 2.In the abstract: (i)Important results from statistical surveys, and results from multivariable logistic regression should be added. (ii)The main results of the demographic statistics (Table 1) that specifically consider three factors: IgG seropositive status, clinical, and epidemiological respectively, should also be included. | 2. (i) DONE  (ii) DONE |
| 3.In the methods: (i)HCW should be associated with the location where they work. It must be mapped what are four hospitals in one city look like, such as the capacity, etc. (related with line 117) | 3. (i)Thank you for your comments, this information is not available. Line 117 has been modified |
| (ii)Statistics methods should be explain more clearly particularly in multivariate logistic regression. Note: Is the multivariable logistic regression same with the multivariate logistic regression?  (iii)Alpha used is too large. It should be 0.01. | (ii)We did NOT use multivariate logistic regression, we used multivariable/multiple logistic regression. Logistic regression formula.  (iii)We applied the standard practice of using alpha 0.05. |
| 4. In the results: (i)Please show the multivariable (multiple) logistic regression model, follows by the estimate parameters, chi-square table, etc. The example of mathematical model is as follows:  (ii)The descriptive statistics is more attractive and interesting if it is represented by figure/graph, for example:  B: Proportion of SARAS-Cov-2 samples across cities and regencies in West Java, Indonesia. C: Distribution of SARS-CoV-2 sequenced samples across different cities and regencies in West Java Indonesia. D: Demography of samples. Source: Azzania, et al. (2021). Analysis of SARS-CoV-2 Genomes from West Java, Indonesia. Viruses (13), 2097. | (i) Below is the model used in the study and we did not see the need to add it in the manuscript.  Univariate logistic regression: The probability function is as below:  p=ⅇ^(β_0+β_1 x)/(1+ⅇ^(β_0+β_1 x) )  Multivariable or Multiple logistic regression: The probability function for multiple explanatory variables is as below:  p=ⅇ^(β_0+β_1 x_1+β_2 x_2+β_3 x_3+⋯.β_n x_n )/(1+e^(β_0+β_1 x_1+β_2 x_2+β_3 x_3+⋯.β_n x_n ) )  (ii) DONE, we added figures of descriptive statistics. |
| (iii)Comparisons should suffice in unrelenting sentences. (What is the comparison table for?) (iv)Demographics should be linked to where they live. | (iii) Thanks for this important comment, the written sentences are enough and the data in the table are more elaborative.  (iv) Thank you for your comment. We don't have location data in our dataset. |
| (v)Add an analysis of the effects of gender on that demographic, i. e. the effect of these results (3 factors: IgG seropositive status, clinical, and epidemiological) on each sex. | (v) Table added |
| Reviewer #2: |  |
| 1.The objective of the study should be clearly stated in the abstract. | 1. The objective has been added. |
| 2. Table 3 (line 186): All the p-values are for the test of significance of the variables not for the regression coefficients. For e.g., there should be a p-value for "occupation", not for certain types of occupation (allied, resp. therapist) - consult your statistician. | 2. We have used occupation in a cross tabulation (chi-square test) in Table 1 but in univariable regression analysis we used dummy variables for each occupational type. Hence, they are statistically sound. |
| 3. Line 193: Fig 1. Trend in mean titer of IgG over the study period. A boxplot is not plotting a mean but a median (Q1, Q2=median, Q3). It would be informative if the titer of IgG are visualized as a line plot per individuals with time and provided by an average line plot for all titer of IgG.  A lowess smoothing plot can be used for the average line plot. Since the individuals are a lot, a soft tone color (grey color, for example) for all individual plot is used. The color for smoothing plot should be bold and strong (black color, for example). An appropriate time scale should be used for the plot, if the date of the serologic tests were available, use "the time since the PCR test" or "the time since the first serologic test" (Choose the best available starting point). Consult your statistician to do such plot. | 3. DONE (Figures added) |
| 4. There is no sound Conclusion (Line 309). There is no answer (at least a comment) to how occupation affect seropositive outcomes (the risk factors), for example. How long the duration of detectable IgG, and how it is as compare to other countries. | 4. Conclusion has been modified and table 6 in discussion demonstrated the differences among countries. |
| 5. Table 1 (Line 163), Characteristics n: there were 5 subjects (2528-273-22650=5) unclassified (seropositive or seronegative). This should be reported either in the table or in the text. | 5. DONE. Stated in the manuscript in abstract and result |
| 6. Be consistent with using or not using a thousand separator (,), for e.g. in line 118: "... a total of 7737 HCWs: 1,021 medical staff. | 6. DONE |
