# Supplementary material for: Seroprevalence and longevity of SARS-CoV-2 nucleocapsid antigen-IgG among health care workers in a large COVID-19 public hospital in Saudi Arabia: A prospective cohort study
Source: PLoS One. 2022 Aug 12;17(8):e0272818. doi: 10.1371/journal.pone.0272818 (PMC9374211; doi:10.1371/journal.pone.0272818)
Supplement: Attachment — Submitted filename: Response to Reviewers.docx [file pone.0272818.s003.docx]

To: Asep K. Supriatna, Ph.D
Academic Editor
PLOS ONE

Re: PONE-D-21-40113R1

Title: Seroprevalence and longevity of SARS-CoV-2 nucleocapsid antigen-IgG among health care workers in a large COVID-19 public hospital in Saudi Arabia: A prospective cohort study

We would like to thank the Editorial Office and the reviewers for their time and valuable comments. We appreciate your efforts in critically reviewing our manuscript and we believe these suggestions would add more depth to our manuscript. We hope we have addressed your concerns appropriately. We look forward to seeing our manuscript published in PLOS ONE. Please find our response to your queries below:

| Academic Editor |  |
| --- | --- |
| 4. Have the authors made all data underlying the findings in their manuscript fully available? | Yes. Submitted in the link |
| Reviewer #2: |  |
| 1. Table 4 is still wrong (previously it was Table 3).  All the p-values should be significance test for the variables not for the regression coefficients. For e.g., there should be a p-value for 'occupation., not for certain types of occupation ('allied', 'resp. therapist', etc.).  The p-value is the simultaneous test for the all dummy variables created by 'occupation' variable. Using this p-value you can evaluate the significance of occupation to the outcome (similar to that of the chi-square test but it is now adjusted for other variables). This is a typical mistake in using regression with dummy variables (with more than 2 categories). Do the same way for all variables with more than 2 categories ('previous medicine', 'medical condition', ... why not include the 'blood group' ...as in the chi-square test?). A sound conclusion then can be made from these p-values: what is the effect of "occupation", what is the effect of "previous medicine", etc.  I suggest you also performing variable selection (model building). In a software like R, there is available procedure like dropterm() to calculate the simultaneous test, stepAIC() for variable selection. It should be available also in STATA, perhaps the TEST syntax. | 1. Thanks for your comment. We have assessed the association between occupation as a whole and serology test (outcome) in Table 1 using chi-square test and found it had a trend to significance (p = 0.08). That's why we tried to test using logistic regression to pinpoint whether any particular occupation is related to serology positivity compared to others. Unfortunately, in logistic regression we can't treat occupation as one single variable/category as it has 8 categories. If we do then Stata will treat it as a continuous variable. Hence, we must declare that it's a categorical variable and must declare which category will be the reference category (usually the category which holds most people (considered to be "normal"). That's why in Stata, we declared occupation to be a categorical variable and declared "Nurse" to be the :reference category" as this category held the highest number of people (n = 1351 (53.0%)) of all participants. Hence, we got OR and p value for all the other occupations compared to Nurses. We have also done model building by taking only those variables from simple logistic regression (unadjusted) that had p values less than 0.3 (either significant or potential to become significant). That's why you will see only a few variables in the multiple logistic regression (adjusted) section. As per your suggestion, we have done model building with variable selection. Please see new table-4 in the manuscript. |
| 2. Fig 2.C  The horizontal axis should be the time, and the vertical axis is the IgG results. If the date of the test available, it would be informative if the time is "the time since the first serologic test" (not just Test 1, Test 2, Test 3). The attached figure is an example of such a plot. The individual plots are for all 185 (?) participants.  The number of days since the first test may not be the same for all participants, even if the measurement time is planned every 3 months, therefore the horizontal axis is not just Test 1, Test 2, Test 3. (in the example, I made day 0 is the baseline and the Test 1 is participants who are underwent the next test (In your data, the baseline is the Test 1).  Fig 2.B and a plot as suggested in the attached file are informative enough. | We thank the reviewer for his valuable suggestion. We have created a new lowess smoothing graph (figure 2-B). |
