# Supplementary material for: Seroprevalence and longevity of SARS-CoV-2 nucleocapsid antigen-IgG among health care workers in a large COVID-19 public hospital in Saudi Arabia: A prospective cohort study
Source: PLoS One. 2022 Aug 12;17(8):e0272818. doi: 10.1371/journal.pone.0272818 (PMC9374211; doi:10.1371/journal.pone.0272818)
Supplement: Attachment — Submitted filename: Response to Reviewers.docx [file pone.0272818.s004.docx]

To: Asep K. Supriatna, Ph.D
Academic Editor
PLOS ONE

Re: PONE-D-21-40113R2

Title: Seroprevalence and longevity of SARS-CoV-2 nucleocapsid antigen-IgG among health care workers in a large COVID-19 public hospital in Saudi Arabia: A prospective cohort study

We would like to thank all the editors and reviewers for the valuable comments. We hope we have addressed all the comments appropriately. We look forward to seeing our manuscript published in PLOS ONE. Please find our response to the journal requirement:

| Journal Requirement |  |
| --- | --- |
| Please review your reference list to ensure that it is complete and correct. If you have cited papers that have been retracted, please include the rationale for doing so in the manuscript text, or remove these references and replace them with relevant current references. Any changes to the reference list should be mentioned in the rebuttal letter that accompanies your revised manuscript. If you need to cite a retracted article, indicate the article’s retracted status in the References list and also include a citation and full reference for the retraction notice. | 1. Thanks for your comment. We have revised the reference list and completed it according to the journal requirement. |
